# Prognostic Value of Circulating sST2 for the Prediction of Mortality in Patients With Cardiac Light-Chain Amyloidosis

**DOI:** 10.3389/fcvm.2020.597472

**Published:** 2021-01-20

**Authors:** Yang Zhang, Ying Xiao, Yongtai Liu, Quan Fang, Zhuang Tian, Jian Li, Daobin Zhou, Zhongpeng Xie, Ruijia Dong, Shuyang Zhang

**Affiliations:** ^1^Department of Cardiology, Peking Union Medical College Hospital, Chinese Academy of Medical Sciences and Peking Union Medical College, Beijing, China; ^2^Department of Hematology, Peking Union Medical College Hospital, Chinese Academy of Medical Sciences and Peking Union Medical College, Beijing, China; ^3^Department of Pathology, Hainan General Hospital, Haikou, China

**Keywords:** soluble ST2, systemic light chain amyloidosis, biomarker, overall survival, heart failure

## Abstract

**Aims:** Systemic light-chain (AL) amyloidosis is a multisystemic disorder leading to multiple organ dysfunction and mortality that is often caused by cardiac involvement. Soluble suppression of tumorigenicity 2 (sST2) is a novel biomarker identified for risk stratification of heart disease. The aim of this study was to investigate the value of circulating sST2 levels in prognosis and mortality risk assessments for the AL amyloidosis population.

**Methods and Results:** A total of 56 patients diagnosed with AL amyloidosis were enrolled in Peking Union Medical College Hospital (PUMCH) from January 2015 to May 2018. The relationships between the clinical parameters and overall survival (OS) and risk factors for disease progression were assessed. Additionally, receiver operating characteristic (ROC) curves, Kaplan–Meier analysis, and Cox hazard models were performed to explore the predictive value of sST2 in mortality rates. We found that the median OS of all patients was 7.3 [interquartile range (IQR) 4.4, 15.9] months. The median baseline sST2 level was 12.2 (IQR 5.1, 31.1) ng/ml, and the sST2 high group had more severe patients with a higher Mayo stage. In the ROC analysis, the area under the curve (AUC) was 0.728 [95% confidence interval (CI) 0.603–0.853] for sST2 to predict the outcomes of AL amyloidosis patients, and the optimal cutoff value was 12.34 ng/ml (sensitivity 80.2%, specificity 61.1%). Moreover, in multivariate Cox proportional hazards regression analysis, sST2 acted as an independent predictor of poor functional outcome in patients with AL amyloidosis.

**Conclusion:** In AL amyloidosis patients, sST2 was a strong and independent prognostic biomarker for all-cause mortality, providing complementary prognostic information of a novel scoring system for risk stratification.

## Introduction

Systemic light-chain (AL) amyloidosis is a rare, multisystemic disorder with an incidence of ~6–7/1,000,000 people (1) and is caused by abnormal production of κ- or λ-type monoclonal light chains in the clonal population of bone marrow plasma cells. Instead of the biological α-helix configuration, these light-chain proteins are misfolded and form a β-pleated sheet. Histology has revealed that amyloid fibrils accumulate and deposit in the extracellular matrix (ECM) of numerous organs, such as the kidney, liver, heart, peripheral nerves, and gland of virtually all organs, interfering with organ function and even leading to death.

Without effective treatment, AL amyloidosis patients only have a median survival of <2 years (2). Since the application of novel chemotherapy agents, the median survival in AL amyloidosis patients has approximately doubled over the past decades. However, no improvement is noted in the first few months after diagnosis, and the prognosis of AL amyloidosis varies tremendously (3). Furthermore, cardiac involvement appears in ~60% of AL amyloidosis patients (4), and it is a major prognostic factor in AL amyloidosis. Tests for serum troponins [i.e., cardiac troponins T (cTnT) or I (cTnI)] and N-terminal pro-brain brain natriuretic peptide (NT-proBNP) are strongly suggested in the 2004 (5) and 2012 revised edition Mayo prognostic staging systems models (6). To improve the prognostic value of the scoring system, some modifications have been suggested, such as introducing new variables, changing the cutoff point, and increasing the sensitivity of detection [high sensitivity (hs)-cTnI or hs-cTnT] (7, 8). In this sense, independent indicators from different pathophysiological pathways could increase the prognostic prediction value of existing scoring systems, which suggests that a novel biomarker could be introduced to improve the algorithm.

ST2 is a member of the interleukin-1 (IL-1) receptor family and has transmembrane (ST2L) and soluble ST2 (sST2) isoforms. sST2 acts as a decoy receptor for IL-33, thereby damaging the binding of IL-33 with ST2L distributed on the cell membrane and thus promoting the activation of intracellular signaling cascades of inflammation. Consequently, cardiac function is impaired (9). The IL-33/ST2 axis plays a pivotal role in cardiovascular diseases, such as heart failure (HF) (10), acute aortic dissection (AAD) (11), and acute myocardial infarction (AMI) (12). In addition, a previous study found (13) that elevated sST2 was implicated in worse cardiovascular outcomes and provided insight into the long-term risk stratification in HF beyond that of other biomarkers. Furthermore, the Translational Initiative on Unique and Novel Strategies for Management of Patients with Heart Failure (TRIUMPH) study (14) revealed a positive correlation between repeated measured sST2 and the risk of all-cause mortality in patients with acute HF. Therefore, the 2017 American College of Cardiology/American Heart Association/Heart Failure Society of America (ACC/AHA/HFSA) update of the HF guidelines (15) has recommended sST2 as a useful biomarker for monitoring and prognosis evaluation.

However, little is known about the predictive value of measured sST2 levels in the prognosis of patients with AL. The aim of the present study was to investigate the role of sST2 in the prognosis and overall survival (OS) assessment and risk stratification in AL amyloidosis patients with cardiac involvement.

## Methods

### Study Population

In brief, hospitalized patients diagnosed with AL amyloidosis without treatment at Peking Union Medical College Hospital (PUMCH) from January 2015 to May 2018 were consecutively enrolled and evaluated. The diagnosis of amyloidosis was confirmed by Congo red-positive fibril deposits found in biopsy, and the AL subtype was identified by different methods, including immunohistochemistry, immunofluorescence, and proteomics, as we previously reported (16). Those patients confirmed with cardiac involvement were included in this prospective and longitudinal cohort study. Blood samples were collected from the patients during hospitalization. Clinical information and laboratory data were also collected.

Cardiac involvement was defined as the mean left ventricular wall thickness on echocardiography >12 mm, and no other cause was found to be NT-proBNP >332 pg/ml in the absence of renal failure or atrial fibrillation (AF) (17). In addition, the heart response was defined as a NT-proBNP response (>30% and >300 pg/ml decrease in patients with baseline NT-proBNP ≥650 pg/ml) or New York Heart Association (NYHA) functional class response (≥2 class decrease in subjects with baseline NYHA class III or IV). Heart progression was defined as NT-proBNP progression (>30% and >300 pg/ml increase), cTn progression (≥33% increase), or left ventricular ejection fraction progression (≥10% decrease) (18). All subjects were followed up for clinical outcomes and adverse events until September 2018. All patients were followed up by outpatient or telephone visits. The study was registered in the National Rare Diseases Registry System (NRDRS) of China (www.nrdrs.org.cn). A total of 56 eligible patients were enrolled and followed up.

### Ethics Statement

Our research was approved by the Ethics Committee of PUMCH and was conducted according to the principles of the Declaration of Helsinki. Informed consent was obtained from the patients for inclusion in the study.

### Measurement of sST2

All blood samples taken from AL amyloidosis patients were collected into a 5-ml vacutainer and centrifuged at 3,000 rpm for 5 min. The serum was stored at −80°C. All samples were analyzed using the same reagent lot. The serum levels of sST2 were measured using a Presage ST2 sandwich ELISA kit (Presage® ST2; Critical Diagnostics, San Diego, CA, USA). The signals were detected by a Labsystems Multiskan MS spectrophotometer (Thermo Labsystems) and calculated using Ascent software v2.6 (Thermo Labsystems). The mean absorbance value (OD450) was measured for standard substances, samples and controls, and the concentration of ST2 (ng/ml) was calculated *via* a standard curve.

### Statistical Analysis

Continuous variables were summarized as means ± standard deviation (SD) or medians (25th, 75th percentiles), and categorical variables were summarized as counts and percentages. Two-sided *P*-values and 95% confidence intervals (CIs) were used in the present study. Differences were considered significant at *P* < 0.05. The distribution of continuous variables was tested for normality using the Kolmogorov–Smirnov test. Categorical variables were analyzed using the chi-square or Fisher's exact tests, and continuous variables were studied using Student's t or Mann–Whitney U tests when appropriate. Non-parametric analysis of variance (Kruskal–Wallis H test) was performed for skewed distributions. OS time was defined as the time from initial treatment to the death time for any cause or the time of last follow-up. Spearman's correlation analysis was performed to compare the correlation between sST2 levels and OS. Cox proportional hazards analysis was used to investigate prognostic factors for mortality rate. The hazard ratio (HR) differed in the ST2 low and high groups, whereas significant variables were entered into the multivariate model. The Kaplan–Meier method was used to describe the survival curves between the low and high sST2 groups (cutoff value 12.34 ng/ml), which were compared using the log-rank test. The log-rank test was used to test whether a difference existed between the survival times of different sST2 groups. A 5-level scoring system was created by incorporating these four variables, sST2, dFLC, troponin I, and NT-proBNP, to predict the OS of patients. Restrictive cubic spline (RCS) modeling was performed using R software (version 3.6.3). Harrell's C-indexes were calculated using the R package survival, and the differences between models were compared using the R package compare C. Receiver operating characteristic (ROC) curve analyses were performed using the R packages survivalROC and risksetROC. The cutoff point was analyzed using the surv_cutpoint of the R package. All data were statistically analyzed using GraphPad Prism 7.0 and SPSS version 21.0 software (SPSS, Chicago, Illinois) and R software (version 3.6.3) by two investigators.

## Results

### Characteristics of Study Subjects

The demographic and clinical characteristics of the 56 subjects are shown in Table 1. We found that the median age was 61 [interquartile range (IQR) 52, 66] years old, and 57.1% of the subjects were male. Forty-six patients were λ light-chain type, whereas 10 patients were κ type in our study. The median level of the difference between the involved and uninvolved free light chain (dFLC) was 278.6 (IQR 123.7, 485.7) mg/dl, and the median concentration of NT-proBNP was 4,010.5 (IQR 2,396.3, 7,227.3) pg/ml. The median level of cTnI was 0.09 μg/L (range 0.04–0.17 μg/L). Additionally, the interventricular septum (IVS), left ventricular posterior wall (LVPW), and left ventricular ejection fraction (LVEF) were 14 (IQR 12, 17) mm, 13 (IQR 11, 15) mm, and 56 (50, 65) mm, respectively. Sixteen of 56 evaluable participants had cardiac progression.

**Table 1:** Comparison of clinical characteristics between patients with low and high sST2 levels.

**Characteristics**	**All patients**	**sST2 low (<12.34 ng/ml)**	**sST2 high (≥12.34 ng/ml)**	***P*-value**
	***N* = 56**	***N* = 29**	***N* = 27**	
Age (years)	61 (52, 66)	62 (53, 69)	58 (50, 66)	0.392^a^
Gender (male, %)	32 (57.14)	14 (48.28)	18 (66.67)	0.165^b^
SBP (mmHg)	105 (95, 119)	107 (96, 121)	103 (94, 117)	0.363^a^
DBP (mmHg)	66 (60, 78)	66 (60, 79)	66 (60, 78)	0.937^a^
Creatinine (mg/dl)	80.0 (69.5, 103.3)	77.0 (67.5, 92.0)	80.0 (71.0, 139.0)	0.061^c^
eGFR (ml/min)	87.3 (70.8, 97.6)	87.0 (71.4, 96.7)	87.6 (70.1, 98.0)	0.772^a^
ALT (U/L)	17.0 (12.0, 25.0)	18.0 (13.3, 26.5)	16.0 (12.0, 18.0)	0.645^a^
ALP (U/L)	83.5 (63.3, 122.8)	76.0 (63.0, 103.5)	109.0 (72.0, 168.0)	0.032^c^
24-Hour urine protein (g)	0.50 (0.13, 2.71)	0.16 (0.10, 2.29)	0.88 (0.30, 2.78)	0.666^c^
dFLC (mg/dl)	278.6 (123.7, 485.7)	298.45 (122.28, 445.14)	271.8 (123.7, 1,019.9)	0.920^c^
cTnI (μg/L)	0.09 (0.04, 0.17)	0.08 (0.05, 0.11)	0.11 (0.04, 0.20)	0.949^c^
NT-proBNP (pg/ml)	4,010.5 (2,396.3, 7,227.3)	3,565 (2,311, 5,179)	4,313 (2,337, 14,675)	0.217^c^
Mayo 2004 stage I/II/II (%)	2/46/52	0/59/41	4/40/56	0.007^d^
Mayo 2012 stage I/II/III/IV (%)	7/22/33/38	7/18/46/29	7/26/19/48	<0.001^d^
IVS (mm)	14 (12–17)	14 (12–17)	13 (12–17)	0.308^c^
LVPW (mm)	13 (11–15)	13 (11–15)	13 (11–15)	0.689^c^
LVEF (%)	56 (50, 65)	59 (50, 66)	55 (53, 63)	0.620^a^
Onset time to diagnosis (months)	10.5 (6.0, 22.8)	10.0 (6.0, 25.0)	13.0 (6.0, 23.0)	0.993^c^
Diagnosis to progress (months)	3.3 (1.4, 7.1)	5.5 (3.1, 9.7)	2.0 (1.2, 5.8)	0.202^c^
Treatment to progress (months)	3.4 (1.0, 9.3)	8.7 (4.3, 412.0)	1.7 (0.7, 4.8)	0.069^c^
OS (months)	7.3 (4.4, 15.9)	8.8 (5.0, 18.4)	5.6 (2.2, 8.2)	0.027^c^
Mortality (death, %)	15 (26.79)	3 (10.3)	12 (44.4)	0.004^b^
sST2 (ng/ml)	12.2 (5.1, 31.1)	5.2 (3.3, 8.9)	31.3 (16.6, 54.5)	<0.001^c^

*SBP, systolic blood pressure; DBP, diastolic blood pressure; eGFR, estimated glomerular filtration rate; ALT, alanine aminotransferase; ALP, alkaline phosphatase; dFLC, difference between the involved and uninvolved free light chain; cTnI, cardiac troponin I; NT-proBNP, N-terminal pro-brain natriuretic peptide; IVS, interventricular septum; LVPW, left ventricular posterior wall; LVEF, left ventricular ejection fraction; OS, overall survival*.

a *Indicates that the t-test for two independent samples was used for analysis*;

b *the results of chi-square test*;

c *the results of non-parametric statistical method*;

d *the results of Mann–Whitney U tests*.

We found that the median sST2 level was 12.2 (IQR 5.1, 31.1) ng/ml, and that all patients were divided into two (low and high) groups based on the cutoff value of sST2 at 12.34 ng/ml. No sex or age differences were noted between the two groups. No significant correlation was found between log(sST2) and age in the analysis (data not shown). No difference in blood pressure, liver function, left ventricular wall thickness, or LVEF was found. Compared with the sST2 low group, dFLC and NT-proBNP were higher in the sST2 high group of AL amyloidosis subjects. Based on the Mayo 2004 staging system, the patients were distributed as follows: 1 (2%) in stage I, 26 (46%) in stage II, and 29 (52%) in stage III. There were 4, 12, 18, and 21 patients in stages I, II, III, and IV, respectively, according to the Mayo 2012 staging system (Table 1). The high sST2 group had more severe AL amyloidosis patients regardless of the classification.

**Figure 1:**
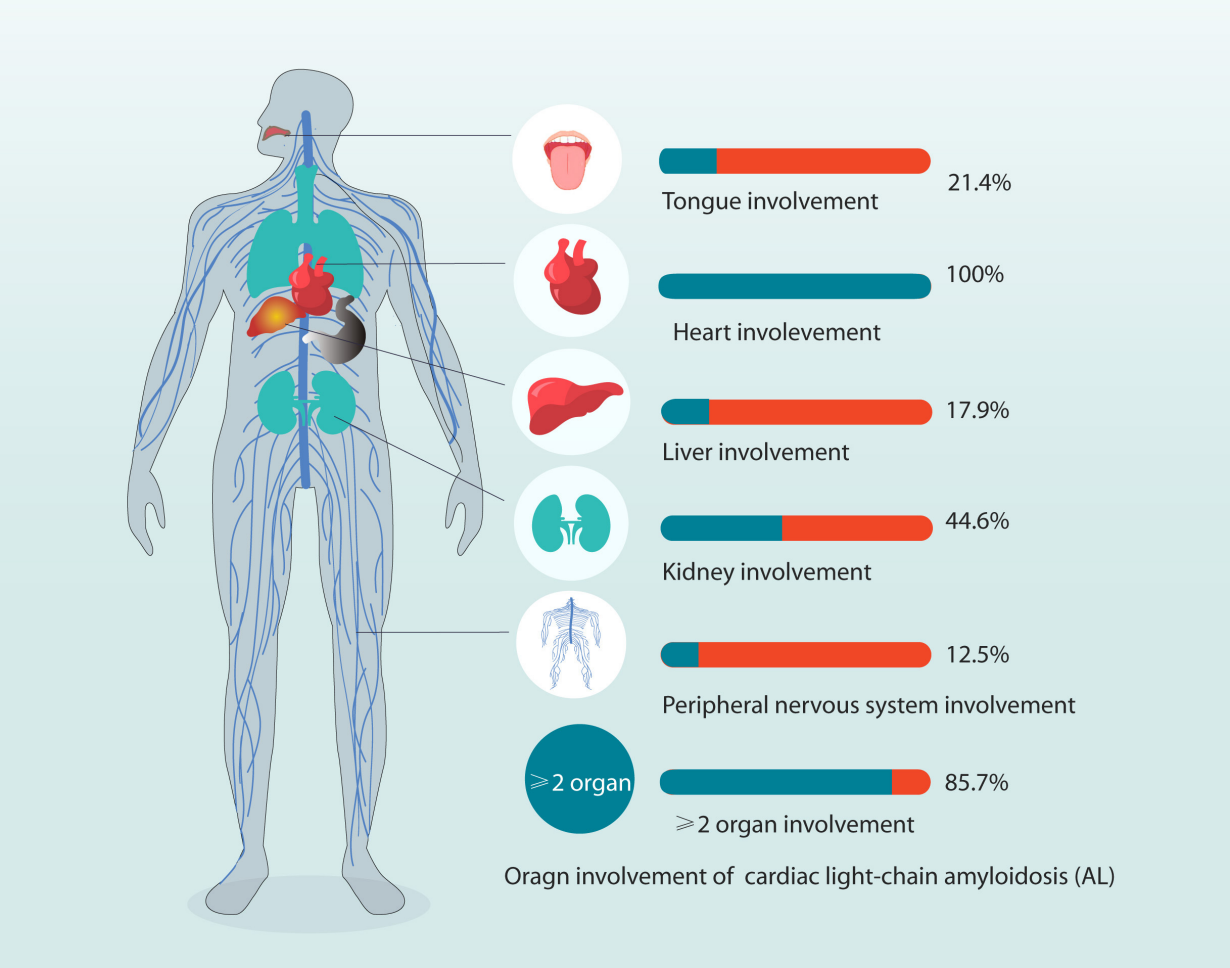
Organ involvement in patients with systemic light-chain amyloidosis in the study. Organ involvement included the heart (100%), kidney (44.6%), liver (17.9%), tongue (21.4%), and PNS (12.5%) (more details in Supplementary Figure 2). In total, 85.7% of patients have at least two organs involved. PNS, peripheral nervous system.

The organ involvements are shown in Figure 1. A majority of patients (85.7%) had more than two organs involved. Organ involvement included the heart (100%), kidney (44.6%), liver (17.9%), tongue (21.4%), and peripheral nervous system (12.5%) (more details in Supplementary Figure 2). The duration time from disease onset to diagnosis was 10 (6.0–22.8) months. Approximately 28.6% of patients progressed after diagnosis, and the median diagnosis to progression time was 3.3 (1.4–7.1) months (Table 1). LVEF <40% was present in 4 of 56 patients (7.1%). All patients had cardiac dysfunction and heart involvement, as evident by predominantly NYHA functional classes III and IV (52%).

### Association Between sST2 and Outcomes of AL Amyloidosis Patients

The median OS was 7.3 months (IQR 4.4, 15.9), and 15 patients died during follow-up. All-cause mortality and disease progression were prospectively adjudicated and verified independently by a local committee every 3–6 months *via* direct patient contact, medical records, and keeping in touch with family members of patients. Patients with sST2 levels <12.34 ng/ml had a substantially lower mortality rate (10.3% vs. 44.4%, *P* = 0.004) and higher survival time [8.8 (IQR 5.0, 18.4) vs. 5.6 (IQR 2.2, 8.2) months, *P* = 0.027, Table 1]. Spearman's correlation analysis showed a statistically significant and negative correlation between sST2 levels and OS (*r* = −0.355, *P* = 0.007).

### The Prognostic Value of Serum sST2 for Future Clinical Events

**Figure 2:**
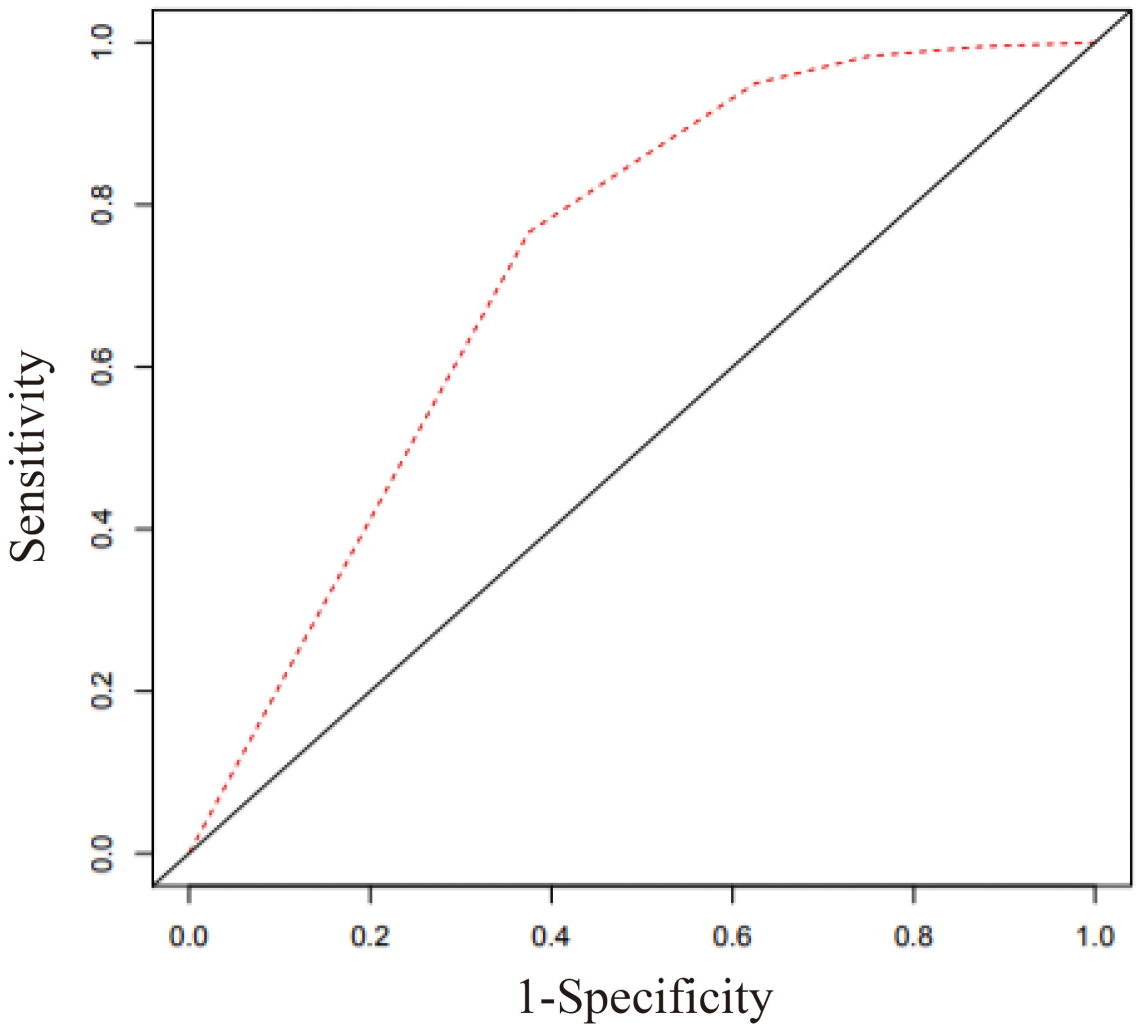
Receiver operating characteristic curve for sST2 based on Cox regression analysis. The optimal cutoff value for sST2 in predicting outcomes of AL amyloidosis was 12.34 ng/ml. The area under the curve was 0.728 (95% CI 0.603–0.853), sensitivity = 80.2%, and specificity = 61.1%.

To explore the predictive value of serum sST2 in the mortality of AL amyloidosis patients, the ROC curve was used, as shown in Figure 2. The area under the curve (AUC) was 0.728 (95% CI 0.603–0.853). At the optimal 12.34 ng/ml cutoff value for serum sST2 based on ROC curve analysis, we found that the sensitivity was 80.2% and the specificity was 61.1% (Youden's index). Additionally, the ROC-derived cutoff point for sST2 to predict 1-year OS was 10.01 ng/ml with an AUC of 0.771 (95% CI 0.638–0.903) and sensitivity and specificity of 91.7 and 54.5%, respectively. Based on the cutoff point of 12.34 ng/ml for sST2, we divided subjects into the low- and high-concentration groups. Strikingly, remarkably significant differences in OS and mortality rates were observed between the two groups (Table 1).

**Figure 3:**
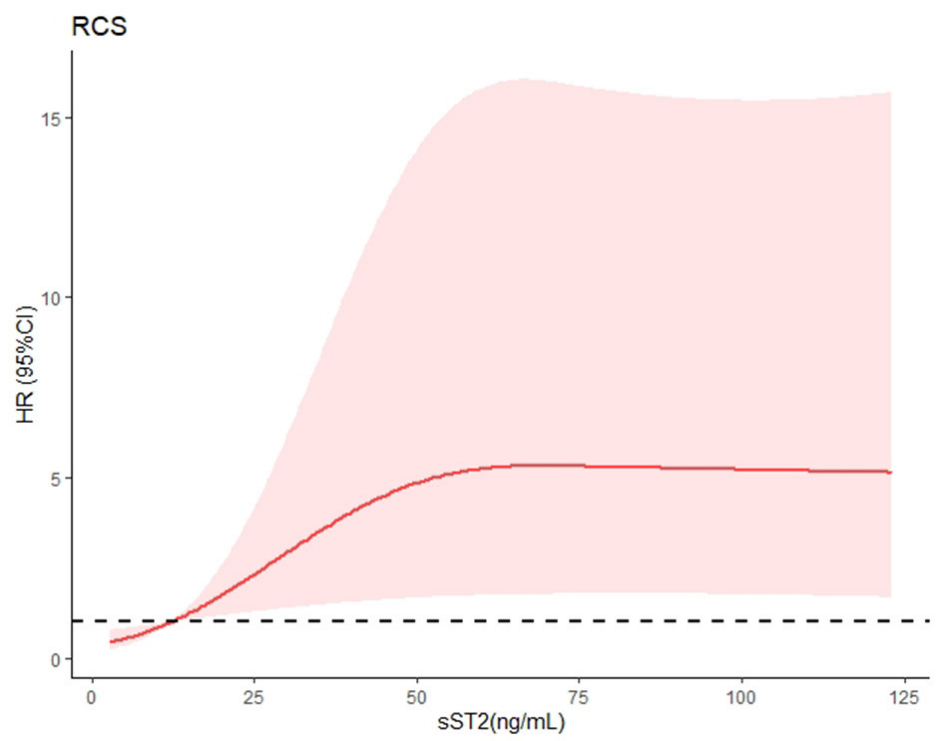
Restrictive cubic spline (RCS) modeling was used to visualize the relationship between sST2 and mortality in AL patients with cardiac involvement. This figure depicts sST2 as a continuous variable in RCS modeling. The risk of mortality increased with increasing sST2 concentrations (*P* for non-linearity <0.01). A broken black line indicates that the hazard ratio equals 1.

**Figure 4:**
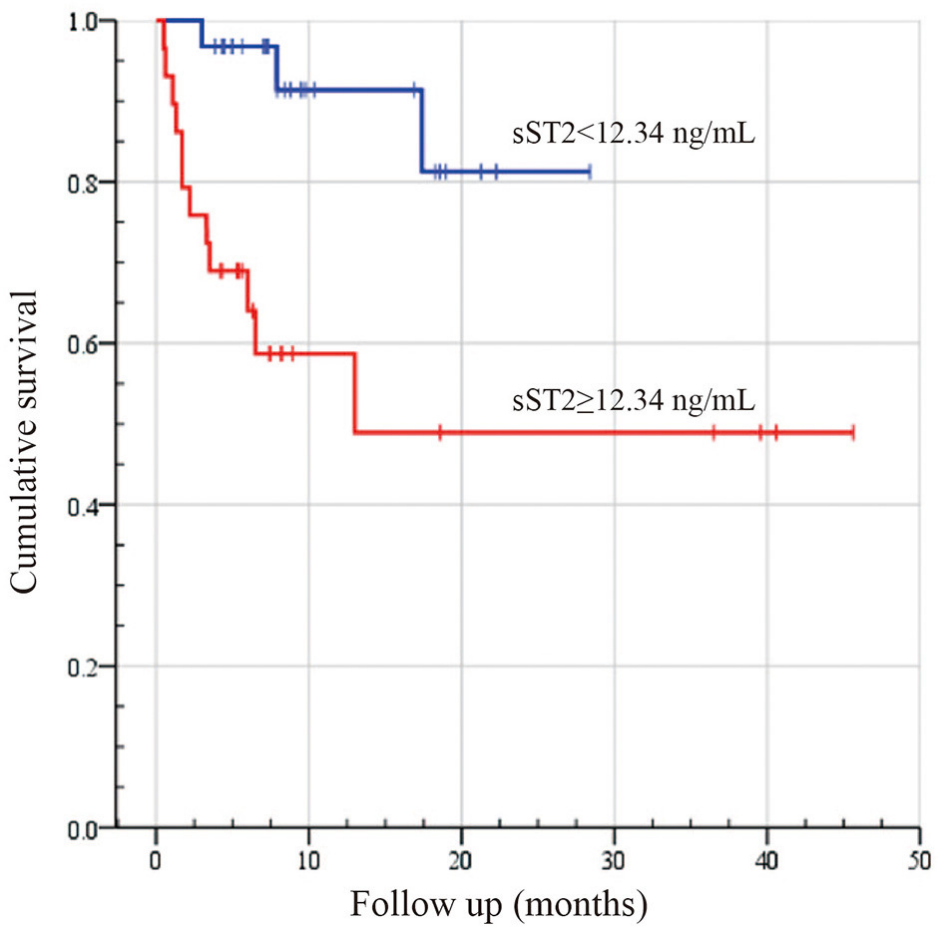
Kaplan–Meier survival analysis between high and low sST2 levels in patients with systemic light-chain amyloidosis. Survival analysis results were compared by employing the log-rank test. Log-rank: *P* < 0.002.

Risk factor screening by univariate analysis was incorporated into the Cox proportional hazards prediction for OS. The analysis results showed that the survival HR of AL patients was significant for sST2 as well as the Mayo 2004 and 2012 staging systems. Because the Mayo 2004 and 2012 staging systems were highly correlated and overlapped, they should not be included in a single multivariable model. Thus, we fitted another three multivariate models in Table 2. Remarkably, the prognostic value of sST2 was independent of the Mayo 2004 and Mayo 2012 staging systems. Then, the Kaplan–Meier method was used to investigate the effects of sST2 levels on the survival of AL amyloidosis patients. Furthermore, we also used RCS modeling with three knots at the 10th, 50th, and 95th centiles to flexibly model and visualize the association of sST2 with mortality. The plot showed a substantial increase in the mortality risk with increasing concentrations of sST2 (*P* for non-linearity <0.01) (Figure 3 and Supplementary Figure 1). Compared with the low level sST2 group (<12.34 ng/ml), the high sST2 group (≥12.34 ng/ml) had a significantly (50% OS was not reached, *P* < 0.001) lower survival time (Figure 4). Harrell's C-index of the existing Mayo 2012 staging system was 0.6840 (95% CI 0.5678–0.8003) with an AUC of 0.702. The C-index of the new prediction model (Mayo 2012 system and sST2) was 0.7850 (95% CI 0.6648–0.9053) with an AUC of 0.794. Statistically significant differences were noted between the two groups (*P* = 0.0038) (Supplementary Figure 3).

**Table 2:** Cox proportional hazards predicting overall survival.

**Models**	**HR**	**95% CI**	***P*-value**
Univariate			
sST2 ≥12.34 ng/ml	5.467	1.515, 19.729	0.009
sST2 concentration (ng/ml)	1.006	1.000, 1.012	0.040
Mayo 2004 stage	3.989	1.240, 12.835	0.020
Mayo 2012 stage	2.370	1.097, 5.117	0.028
Multivariate, 1			
sST2 ≥12.34 ng/ml	4.866	1.341, 17.652	0.016
Mayo 2004 stage	3.379	1.061, 10.760	0.039
Multivariate, 2			
sST2 ≥12.34 ng/ml	4.866	1.341, 17.652	0.016
Mayo 2012 stage	3.379	1.061, 10.760	0.039
Multivariate, 3			
sST2 concentration (ng/ml)	1.009	1.002, 1.016	0.012
Mayo 2012 stage	2.447	1.210, 4.950	0.013

*Mayo 2004 stage incorporates cTnI/cTnT and NT-proBNP*.

*The Mayo 2012 stage system includes cTnI/cTnT, NT-proBNP, and dFLC*.

**Figure 5:**
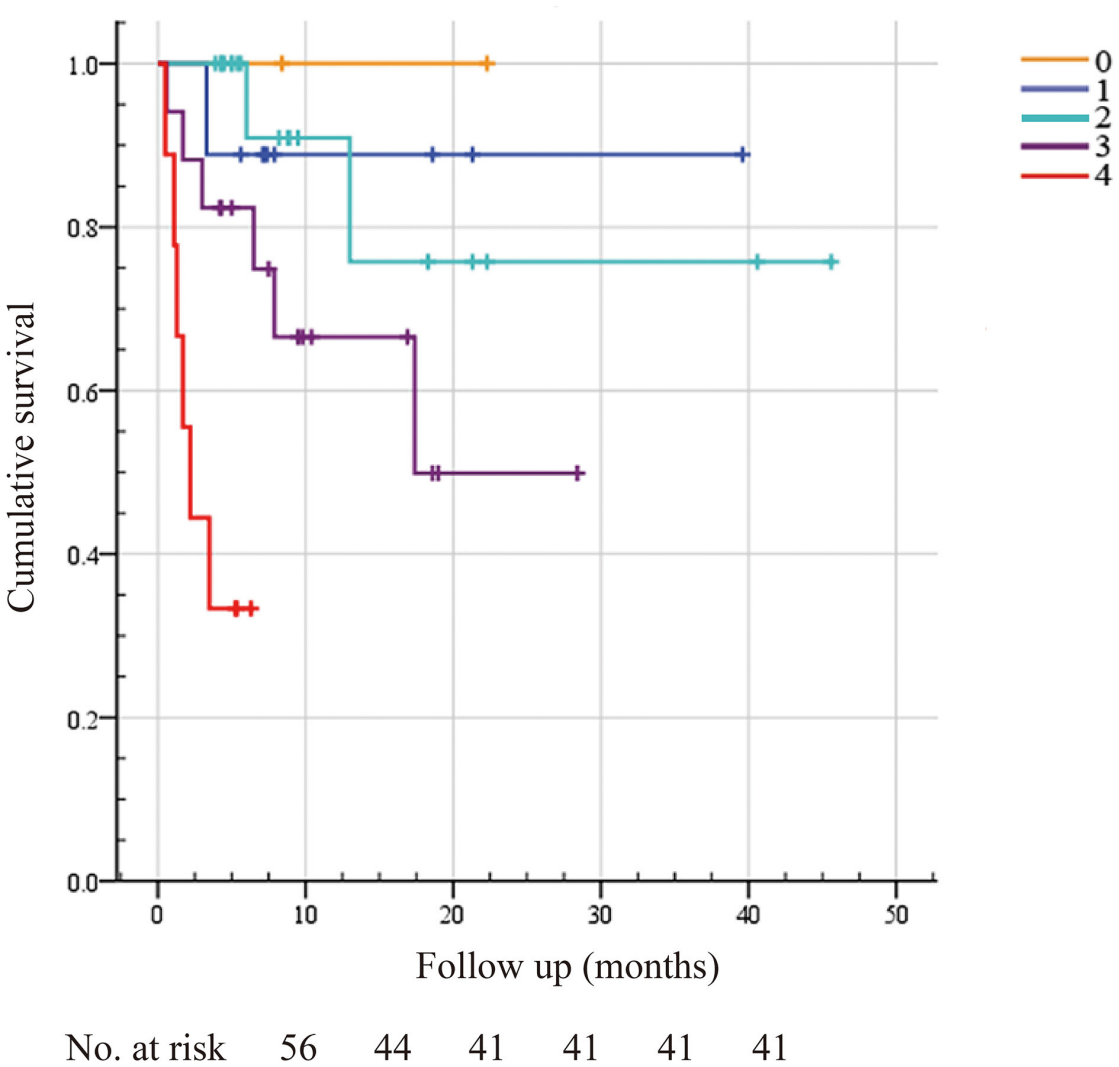
A new scoring system for predicting overall survival incorporating sST2, troponin I, NT-proBNP, and dFLC. A new scoring system including ST2 and other known cardiac risk factors, such as the dFLC and Mayo 2012 staging systems, was built to predict overall survival. We assigned 1 point for sST2 ≥12.34 ng/ml, troponin I >0.025 μg/L, NT-proBNP >1,800 pg/ml, and dFLC >180 mg/L, as reported before. The maximum score was 4, and the minimum score was 0. Score 0, none of the four conditions are met; Score 1, one condition is met; Score 2, two conditions are met; Score 3, three conditions are met; Score 4, all four conditions are met. There were 0 (2), 1 (9), 2 (19), 6 (17), and 6 (9) death events in the 0–4 scoring system, respectively.

Therefore, combining the other three known risk factors in the Mayo 2012 staging system (cTnI, NT-proBNP, dFLC) with sST2 at a value of 12.34 ng/ml, a new scoring system was established for predicting the OS of AL amyloidosis patients as reported previously (6). Incorporating these variables, a 5-level scoring system was created, as shown in Figure 5. There were 0 (2), 1 (9), 2 (19), 6 (17), and 6 (9) deaths with the 0–4 scoring system, respectively. Moreover, the HR per level in our system was 3.932 (95% CI 1.82–8.49, *P* < 0.001). Compared with the Mayo 2012 staging system [AUC 0.683 (95% CI 0.523–0.843), sensitivity = 66.7%, specificity = 70.7%], our new scoring system [AUC 0.772 (95% CI 0.630–0.915), sensitivity = 80.0%, specificity = 65.9%] could better predict OS (*P* < 0.05).

## Discussion

As cardiac involvement is a major determinant for treatment and prognosis in AL amyloidosis, scoring systems based on biomarkers of different pathophysiological pathways have been progressively elucidated. Here, our data showed that sST2 was a novel and independent prognostic factor for patients with AL amyloidosis.

To optimize treatments and improve OS in AL amyloidosis patients, it is necessary to build a prognostic evaluation system based on target organ damage and plasma cell dyscrasia. In this respect, the Mayo 2004 or 2012 staging systems to estimate cardiac involvement and long-term survival are widely used. cTnT(I) is related to myocardial necrosis, and NT-proBNP reflects myocardial motion. Both cTnT(I) and NT-proBNP are important serum cardiac biomarkers in the current evaluation system. Moreover, emerging studies have revealed that introducing new variables could improve the predictive value of the staging system (19, 20). RCS modeling indicated a significant nonlinear correlation (*P* < 0.01) between sST2 concentrations and mortality rate, which increased with sST2 levels. However, HR did not increase significantly with sST2 levels exceeding 60 ng/ml, which was probably caused by the limited sample size. Multicenter clinical studies may be needed in the future. sST2 is a novel powerful and widely proven valuable biomarker that reflects the degree of cardiac fibrosis and remodeling. A study of 346 patients with acute HF showed that the concentrations of sST2 represented the severity of disease and powerfully portended a negative prognosis (21). High sST2 levels were strongly predictive of mortality at 1 year in patients with acute dyspnea (22) regardless of acute HF. In addition, increased sST2 levels predict all-cause mortality in patients with acute coronary syndrome (ACS) (23) and can be used as a risk factor for stratification in patients with ST elevation acute myocardial infarction (STEMI) (24). Similarly, we revealed that the prognostic value of sST2 was independent of the existing Mayo staging systems in patients with AL amyloidosis and a new scoring system was established in the present study.

The number and severity of organ involvement determines the prognosis in AL amyloidosis patients, ~60% of whom have cardiac involvement, which carries a poor prognosis (25, 26). Consistently, we found that the median OS times in our subjects were lower than those previously reported (3, 4) because all of the patients in our study have cardiac involvement to some extent. Soluble cardiac biomarkers, such as NT-proBNP, cTnI, or cTnT, can help detect cardiac involvement early and improve outcomes through effective interventions. To date, the mechanism of cardiac involvement in AL amyloidosis remains unclear (27). However, an increasing number of studies have revealed that redox stress, cellular dysfunction, and apoptosis contribute to myocardial damage and subsequent HF (28, 29). Limited to the invasiveness and complexity of endomyocardial biopsy, soluble biomarkers of the circulatory system could dynamically monitor changes in cardiac function. Unlike almost any other biomarker currently used, sST2 is minimally affected by age, sex, body mass index (BMI), AF, and even renal function. Due to minimal interindividual variability, horizontal comparisons are ideal. Moreover, sST2 levels can be used not only for risk assessment during hospitalization of HF patients but also for prognosis prediction after discharge. Therefore, the addition of sST2 to the Mayo scoring system can improve its stability and accuracy; in fact, we have confirmed that the new algorithm had better predictive value.

The most appropriate cutoff value for sST2 was reported to be different in the general population (30) and in the prediction of outcomes for different diseases. Indeed, the optimal cutoff value of sST2 (12.34 ng/ml) in our study based on the ROC curve was lower than that reported by other studies (11, 31). Similarly, low concentrations of sST2 (13.5 ng/ml) could act as a biomarker for detecting stable HF with a normal ejection fraction (HFNEF) in hypertensive patients (32). The cutoff value of 12.34 ng/ml was taken into consideration for the study due to the distinct disease characteristics. On the one hand, as a rare disease, the small number of subjects might introduce biases into the results despite statistical adjustment. Although derived from different diseases, our subsequent analysis did confirm that the investigated cutoff value of sST2 may be more suitable for prediction in the new scoring system for AL amyloidosis. In the future, we will conduct large sample and multicenter studies to reduce this bias. On the other hand, only patients confirmed with cardiac involvement were included in our prospective and longitudinal cohort study of AL amyloidosis. For diseases with unknown causes of myocardial damage, sST2 concentrations may be completely different.

### Limitations

There were some limitations in our study. First, although we analyzed different concentrations of sST2, the small number of patients may limit our ability to examine sST2's utility in AL amyloidosis; thus, the calculated cutoff value of sST2 (12.34 ng/ml) may be more suitable and was adopted for further analysis. However, to our knowledge, there are still no large-sample studies targeting sST2 to investigate the long-term prognosis of AL amyloidosis with cardiac involvement. Second, the follow-up period of some patients was very short, and continuous follow-up and multicenter clinical studies are needed in the future.

## Conclusions

In conclusion, we identified that sST2 was a strong and independent prognostic biomarker for OS and all-cause mortality in patients with AL amyloidosis. Moreover, a novel scoring system consisting of sST2 may be used for risk stratification and prognosis prediction in AL amyloidosis patients.

## Data Availability Statement

The raw data supporting the conclusions of this article will be made available by the authors, without undue reservation.

## Ethics Statement

The studies involving human participants were reviewed and approved by Ethics Committee of Peking Union Medical College Hospital. The patients/participants provided their written informed consent to participate in this study.

## Author Contributions

YZ and YX contributed to the conceptualization, data curation, writing, original draft preparation, and visualization of this study. YL, QF, and DZ contributed to the formal examination. ZX and RD helped performed the statistical tests. ZT, JL, and SZ acquired the funding and contributed to the supervision and editing of the review. All authors contributed to the article and approved the submitted version.

## Conflict of Interest

The authors declare that the research was conducted in the absence of any commercial or financial relationships that could be construed as a potential conflict of interest.
