# Supplementary material for: Prognostic Value of Circulating sST2 for the Prediction of Mortality in Patients With Cardiac Light-Chain Amyloidosis
Source: Front Cardiovasc Med. 2021 Jan 20;7:597472. doi: 10.3389/fcvm.2020.597472 (PMC7855859; doi:10.3389/fcvm.2020.597472)
Supplement: Supplementary file 1 [file Data_Sheet_1.pdf]

Figure S1. Restrictive cubic spline (RCS) modeling was used to visualize the relationship between sST2 and mortality in AL patients with cardiac involvement.

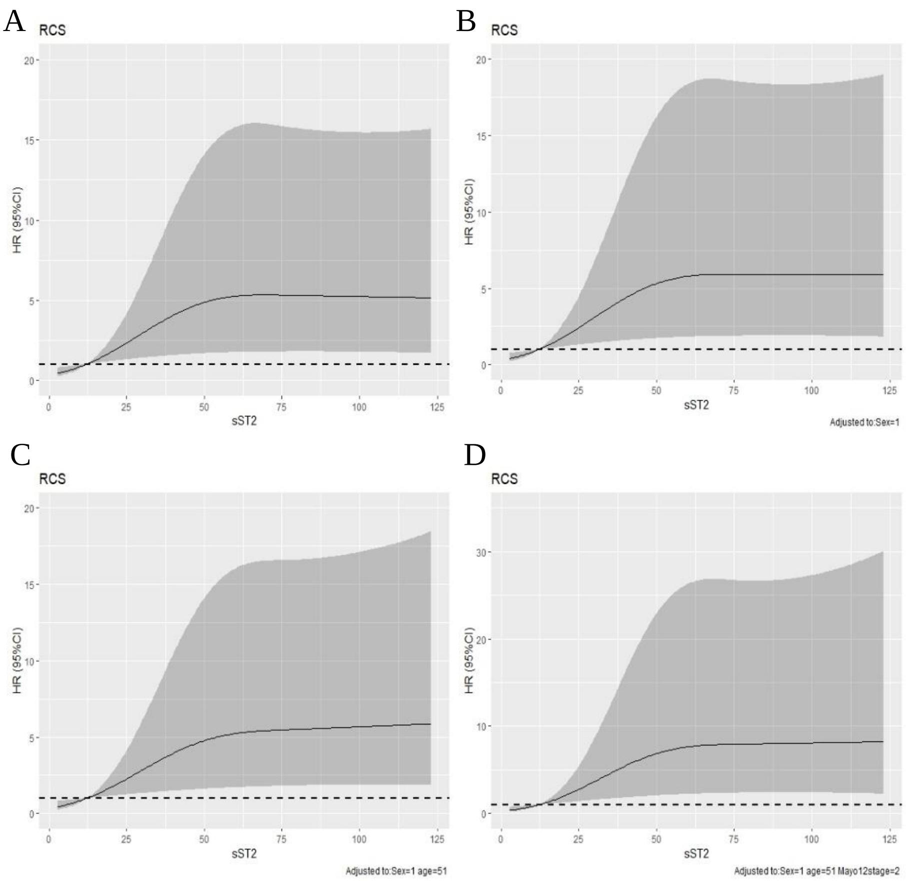

Picture A (model A) presents sST2 as a continuous variable in restrictive cubic spline modeling. Picture B represents model B after adjusting for sex. Model C was adjusted for sex and age, and model D was adjusted for sex, age and the Mayo 2012 staging system. We selected 3 knots at the 10th, 50th, and 95th percentiles in the models. The plot showed a substantial increase in the mortality rate with increasing concentrations of sST2 (P for nonlinearity =0.0012 < 0.05).

Figure S2. The number of organs involved among different serum sST2 concentration groups.

No. of patients	sST2 group (12.34 ng/mL)	Cardiac involvement	Kidney involvement	Liver involvement	Peripheral nervous system	Tongue involvement	Pleural effusion	Gastrointestinal involvement	No. of organs involved
01	Low	*							1
02	Low	*			◆	▲			3
03	Low	*					●		2
04	Low	*					●		2
05	Low	*	▽						2
06	Low	*	▽						2
07	Low	*							1
08	Low	*	▽	◇			●		4
09	Low	*				▲			2
10	Low	*			◆				2
11	Low	*				▲			2
12	Low	*	▽						2
13	Low	*				▲	●		3
14	Low	*							1
15	Low	*						#	2
16	Low	*	▽				●		3
17	Low	*	▽					#	3
18	Low	*	▽			▲			3
19	Low	*					●		2
20	Low	*							1

21	Low	*	▽						2
22	Low	*		◇		▲	●		4
23	Low	*				▲	●		3
24	Low	*							1
25	Low	*				▲			2
26	Low	*	▽		◆				3
27	Low	*		◇			●		3
28	Low	*	▽						2
29	Low	*				▲	●		3
30	High	*	▽				●		3
31	High	*					●		2
32	High	*				▲	●		3
33	High	*	▽				●		3
34	High	*	▽		◆	▲	●		5
35	High	*	▽						2
36	High	*					●		2
37	High	*							1
38	High	*	▽	◇					3
39	High	*	▽		◆			#	4
40	High	*	▽	◇	◆		●		5
41	High	*					●		2
42	High	*	▽						2
43	High	*							1
44	High	*					●		2
45	High	*	▽	◇					3
46	High	*	▽				●		3

47	High	*	▽				●		3
48	High	*					●		2
49	High	*	▽						2
50	High	*	▽	◇					3
51	High	*	▽						2
52	High	*	▽				●		3
53	High	*		◇					2
54	High	*			◆	▲	●	#	5
55	High	*	▽	◇					2
56	High	*		◇					1

Table. No statistically significant difference regarding the number of organs involved was noted between the two sST2 groups (*P*>0.05). Patients with sST2 < 12.34 ng/mL were defined as the low sST2 group, whereas the high sST2 group was defined as the ≥ 12.34 ng/mL group.

Figure S3. ROC curve based on Cox regression analysis to predict the mortality rate of AL amyloidosis patients with cardiac involvement.

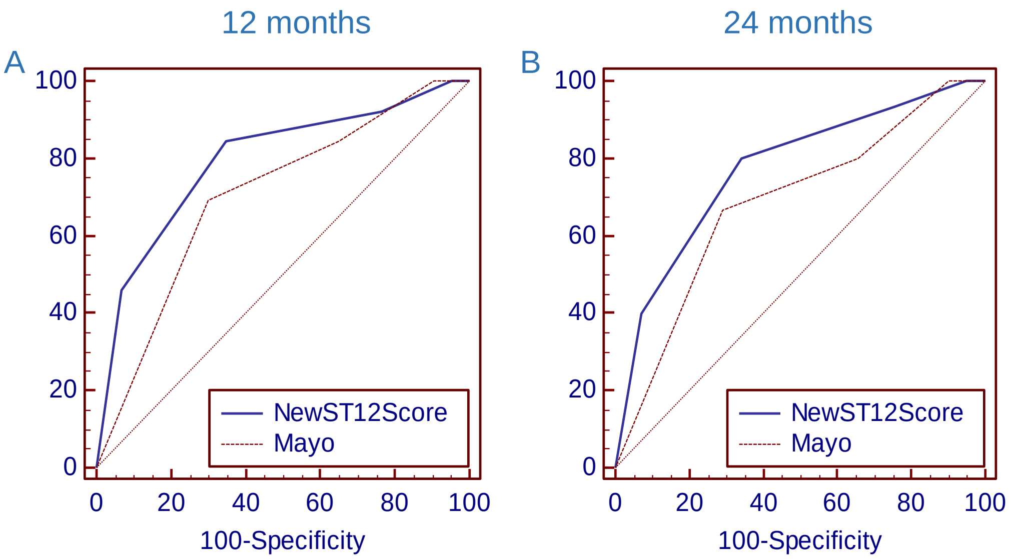

The analyses were performed using the R software. Receiver operating characteristic (ROC) curve analyses were performed using the R package survivalROC. Harrell's C-index of the existing Mayo2012 staging system was 0.6840 (95% CI 0.5678-0.8003) with an AUC of 0.702. The C-index of the new prediction model (Mayo2012 system+sST2) was 0.7850 (95% CI 0.6648-0.9053) with an AUC of 0.794. Statistically significant differences were noted between the two groups (P=0.0038). Model A predicted the disease outcomes at 12 months, and model B predicted the disease outcomes at 24 months.
